# The effect of aeration and irrigation on the improvement of soil environment and yield in dryland maize

**DOI:** 10.3389/fpls.2024.1464624

**Published:** 2024-10-18

**Authors:** Zhen-zhen Yu, Hong-xuan Wang, De-shui Yu, Ning-xia Yin, Jing Zhang

**Affiliations:** ^1^ School of Mechanical Engineering, Guangdong Ocean University, Zhanjiang, China; ^2^ South Subtropical Crops Research Institute, Chinese Academy of Tropical Agricultural Sciences, Key Laboratory of Tropical Fruit Biology, Ministry of Agriculture & Rural Affairs, Zhanjiang, China; ^3^ School of Management, Huazhong University of Science and Technology, Wuhan, China

**Keywords:** aerated irrigation, dryland maize, soil environment, structural equation model, mechanisms of yield increase

## Abstract

The aim of this study was to examine the effect of long-term aerated seepage irrigation technology on soil fertility changes and maize yield under continuous maize cropping system in red loam soil, and to explain the mechanism of maize yield increase under this technology, which can provide theoretical basis for crop quality improvement and yield increase under aerated irrigation (AI) technology. Therefore, this research was conducted for four field seasons in 2020–2023 at the National Soil Quality Observation Experimental Station, Zhanjiang, China. Soil aeration, soil fertility, root growth, physiological traits, and yield indicators were evaluated by conventional underground drip irrigation (CK) and AI. Our results showed that AI treatment significantly improved soil aeration and soil fertility. Increases in soil oxygen content, soil respiration rate, soil bacterial biomass, and soil urease activity were observed, corresponding to increases from 3.08% to 21.34%, 1.90% to 24.71%, 26.37% to 0.09%, and 12.35% to 100.96%, respectively. The effect of AI on maize indicators increased year by year. Based on improvements in soil aeration and fertility, root length, root surface area, and root dry weight under AI treatment were enhanced by 15.56% to 53.79%, 30.13% to 62.31%, and 19.23% to 35.64% (*p* < 0.05) compared to the CK group. In addition, maize agronomic traits and physiological characteristics showed improved performance; in particular, over 1.16% to 14.42% increases were identified in maize yield by AI treatment. Further analysis using a structural equation model (SEM) demonstrated that the AI technology significantly promotes the improvement of root indicators by enhancing soil aeration and soil fertility. As a result, maize yield could be increased significantly and indirectly

## 1 Introduction

Water, fertilizer, air, and heat in the soil are the four major factors that safeguard soil fertility (Yu et al., 2022b), and prolonged flooding or over-irrigation conditions are prone to low-oxygen stress in the root zone of crops (Shahzad et al., 2019; Wei et al., 2021). In recent years, with the rapid development of facility agriculture, anthropogenic factors such as over-irrigation, crushing by agricultural machinery, over-fertilization, and less mid-tillage may all lead to soil compacting (Xiao R. et al., 2023), reducing soil porosity, soil aeration, and fertility, resulting in weakened soil microbial activity, impeded root respiration (Duan. et al., 2024; Li et al., 2024; Mondal et al., 2024), and reduced water and fertilizer uptake, and, in severe cases, leading to the development of physiological diseases of the root system, such as root rot (Xiao Z. et al., 2023), further weakening the absorption function of the root system, reducing the growth rate and yield of crops, and affecting the overall health of crops and the quality of agricultural products (Ouyang and Tian, 2023; Wei et al., 2021; Zhang et al., 2023). Especially in southern red loam soils, where the soil texture is more clayey and poorly drained and prone to waterlogging, the problem of low oxygen stress in the root zone is more prominent. Therefore, optimizing soil moisture management and irrigation strategies to improve soil oxygen supply is important for improving soil oxygen supply, crop root health, and growth in southern red loam soils.

In 1949, Melsted (Melsted et al., 1949) first began experimental research on soil aeration and oxygenation in the crop root zone, and in recent years, it has gradually developed into diversified aeration and percolation irrigation technology models (mechanical aeration irrigation, chemical aeration irrigation, and Venturi air jet irrigator irrigation), and experimental studies have been carried out for different geographic regions, different crops, and different soil types. The results of a large number of studies have shown that aerated infiltration technology enhances soil respiration by improving soil aeration, increasing soil oxygen content (Yu, 2020; Yu et al., 2022a; Li R. et al., 2023; DeBoer et al., 2024), and enhancing soil respiration (Pang et al., 2023), and has a positive impact on a wide range of greenhouse tomatoes (Wei et al., 2021; Xiao Z. et al., 2023; Li et al., 2024), cucumbers (Ouyang and Tian, 2023; Zhang et al., 2023), maize (Melsted et al., 1949; Yu, 2020; Yu et al., 2022a; Li R. et al., 2023), melons (Pang et al., 2023; DeBoer et al., 2024), watermelons (Ouyang, 2018), grapes (Zhao et al., 2017), and chili peppers (Lei et al., 2023a), among other crops under cultivation that have positively influenced yield and quality. Abuarab et al. conducted field trials on greenhouse tomato, potato, maize, melon, and cotton through mechanically aerated irrigation (AI), and the results showed that aerated compared to unaerated treatments significantly enhanced soil respiration rate and soil oxygen content at different fertility stages, and demonstrated that AI mainly improves soil respiration rate by increasing the soil oxygen content in the root zone and thereby increasing the soil respiration rate (Abuarab et al., 2013; Zhou et al., 2023; Zhuang et al., 2024). Lei et al. showed that the root zone aeration after irrigation soil oxygen diffusion rate and redox potential increased significantly, and sufficient oxygen for the normal metabolism of aerobic microorganisms and energy production provides a good living environment, enhances the number of soil bacteria and fungi, improves soil enzyme activity, and promotes microbial respiration to complete the decomposition of the substrate and synthesis of cellular material and biochemical reaction rate, while avoiding the anaerobic environment. The accumulation of harmful metabolites (e.g., lactic acid and ethanol) commonly found in anaerobic environments (Zhu, 2020; Lei et al., 2023b; Lian, 2023; Zhang et al., 2024; Zhuang et al., 2024) realizes soil nutrient cycling and transformation and promotes the growth and development of the root system, which can improve the morphology of the root system, enhance the root system vitality, and promote the efficiency of the crop root system in absorbing water and nutrients. The study of Bhattarai and other studies involving experiments on potted vegetables, field maize, soybean, and pumpkin cultivation, crops under AI treatments, showed accelerated growth, increased leaf thickness, elevated chlorophyll content, and improved photosynthetic efficiency, which ultimately manifested in increased yields and improved quality (Bhattarai et al., 2008; Palada et al., 2010; Silwal et al., 2020; Yu et al., 2022a; Yu, 2023; Xu et al., 2024).

Although a large number of studies have proved the effect of aerated infiltration technology in improving the soil environment and crop yield and quality, owing to the superimposed effect between soil and crop indicators under aerated infiltration technology, for example, the improvement of soil oxygen content not only directly promotes root respiration, but also indirectly affects nutrient decomposition and uptake through the improvement of soil microbial activity, and this complex interaction makes it difficult to accurately quantify the independent contribution of each factor to yield and quality in existing studies. The key factors and pathways used by aerated percolation technology to drive crop yield and quality improvement on the basis of improving the soil environment still need to be thoroughly explored and researched.

Based on this, our study at the National Soil Quality Zhanjiang Observation and Experimental Station utilized a 4-year aeration irrigation positioning experiment to analyze the effects of long-term aeration irrigation technology on soil environmental changes and maize yield during the seedling stage (VE), jointing stage (V6), tasseling stage (VT), grain filling stage (R2), and maturity stage (R5). By constructing a soil-crop growth structural equation model under the aeration irrigation mode and using path analysis, we aimed to elucidate the mechanisms and regulatory pathways of crop yield improvement under aeration irrigation technology. The results of the study provide theoretical basis and practical reference in supplementing and perfecting the mechanism of crop yield increase under aeration irrigation technology, promoting and applying the technology, and at the same time, it can provide reference for adjusting the implementation scheme of aeration irrigation in the experimental background of different regions, and realizing the application and promotion of the technology in different regions.

## 2 Materials and methods

### 2.1 Experimental site

**Figure 1:**
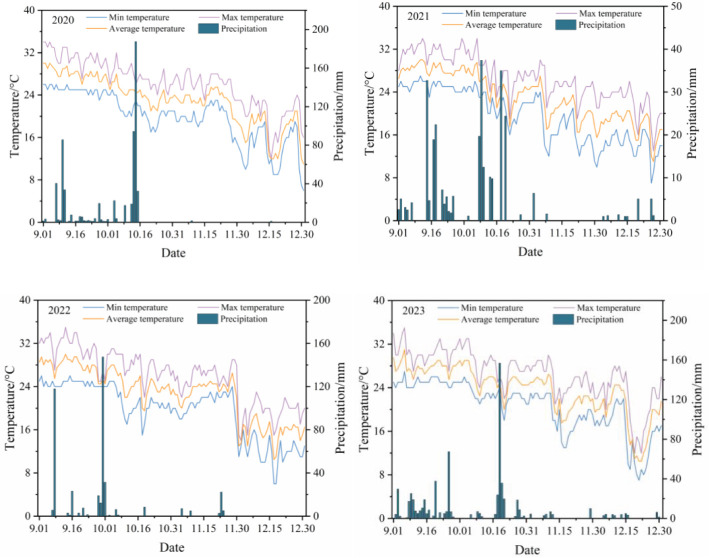
Variation curves of mean air temperature and rainfall during the test period from 2020 to 2023.

The experiment was conducted at the National Soil Quality Zhanjiang Observation Experiment Station of the Chinese Academy of Tropical Agricultural Sciences in Zhanjiang, Guangdong, China (E109°31′, N21°35′). The experiment was conducted in plots with a burial depth of 20 cm, a diameter of 16 mm, a flow rate of 2.5 L/h, and a drip head spacing of 20 cm in 2019. The experimental period was from September 2020 to January 2024. The experimental site has a typical subtropical monsoon climate, with an annual sunshine time of 1900–2100 h, an annual frost-free period of more than 350 days, and an annual average temperature of 23.5°C. Rainfall, air temperature, and other environmental factors during the experimental period were automatically obtained and recorded by micro weather stations in the experiment site (**Figure 1**).

### 2.2 Experimental design and methods

**Figure 2:**
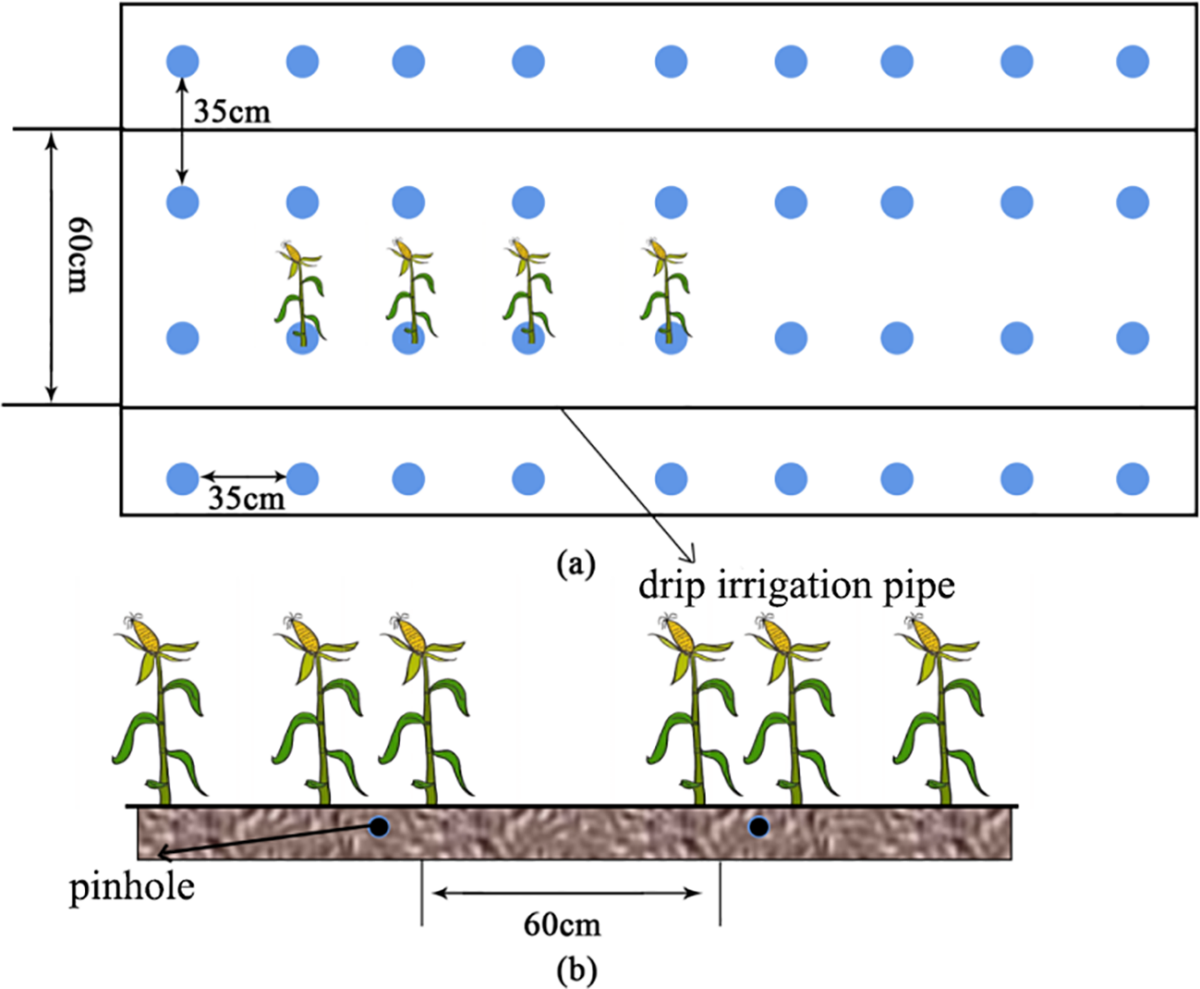
Vertical view **(A)** and front view **(B)** of aerated irrigation maize planting patterns.

The maize variety planted in the experimental area was “Huiyu Sweet No. 3”, with a fertility period of 120–150 days. The maize was planted in fall and winter every year and was planted at the beginning of September and harvested at the end of December or the beginning of January. The maize planting parameters are as shown in **Figure 2**, with a planting pattern of two tubes and four rows and a planting density of 14,400 plants/ha. A Roots fan (HRE65WA, pressure set at 0.7 MPa) was connected to the dry pipe and aerated after irrigation or rainfall. The experiment was conducted with two treatments: conventional underground drip irrigation (CK) and aeration irrigation (AI). Each treatment had three experimental plots with each plot measuring 12.5 m² (5 m × 2.5 m). Fertilization measurements were consistent across different treatments. There were three experimental plots for each treatment, and each plot was 12.5 m² (5 m × 2.5 m) in size. Each experimental unit was surrounded by one protection row. The same field management practices were applied to each treatment with a basal fertilizer of 36 kg/hm^2^ of nitrogen before sowing; 75 kg/hm^2^ of P_2_O_5_ and 37.5 kg/hm^2^ of K_2_O were applied before sowing. Phosphorus and potash fertilizers were applied in the same rate during the growth period. Standard pests and weed control were performed according to maize growing guidance. During the maize growth cycle, measurements were taken approximately every 10 days, with delays if heavy rainfall occurred. For each test, the average values of maize at different growth stages were statistically analyzed, including the seedling stage (VE), jointing stage (V6), tasseling stage (VT), grain filling stage (R2), and maturity stage (R5).

The irrigation amount for the experiment was determined using the crop-pan coefficient method. The evaporation amount measured by the standard E601 evaporation pan was used to control the irrigation amount. The irrigation time was 08:00–12:00 or 16:00–18:00, and the period was 3–4 days. The amount of water was based on the evaporation measured at 08:00 in the morning of each day during the irrigation interval. The calculation of the irrigation amount is shown in Equation 1 (Yu, 2020):


(1) $$W=A\cdot E_{P}\cdot K_{P}$$

In the formula, *W* represents the irrigation amount per treatment per event, in liters (L). *A* is the area controlled by a single dripper, which is 0.14 m² (0.35 m × 0.4 m), *E*_p_ is the evaporation amount measured by the evaporation pan between the time intervals between two irrigation events, in millimeters (mm), and *K*_p_ is the crop-pan coefficient. For maize, the *K*_p_ values are 0.6 during the VE–V6 stage, 0.8 during the V6–VT stage, 1.0 during the VT–R2 stage, and 0.8 during the R2–R5 stage (Yu, 2020).

Soil aeration was carried out throughout the reproductive cycle of maize, which was aerated at a frequency of 1 in 2 days, and the aeration volume was calculated by Equation 2 as (Melsted et al., 1949), and the escape of gas from the soil was not considered in the experiment.


(2) $$V=1/1000SL(1-\rho_{b}/\rho_{s})$$

In the equation, *V* represents the amount of aeration per session in liters (L); *S* is the cross-sectional area of the ridge, 1,500 cm², *L* is the length of the ridge in meters (m), 550 cm, $\rho_{\text{s}}$ is the soil bulk density, 1.62 g/cm^3^, $\rho_{\text{b}}$ is the soil density, and the mean value of the density of 0–100 cm soil determined by the ring knife method was 2.67 g/cm^3^. According to the actual planting area of the plot, the aeration volume is calculated as 324.6 L. Aerating was done once a day between 17:00 and 19:00, and the escape of gas from the soil was not considered in the experiment.

### 2.3 Measurement indicators and methods

#### 2.3.1 Soil aeration

##### 2.3.1.1 Soil respiration rate

**Figure 3:**
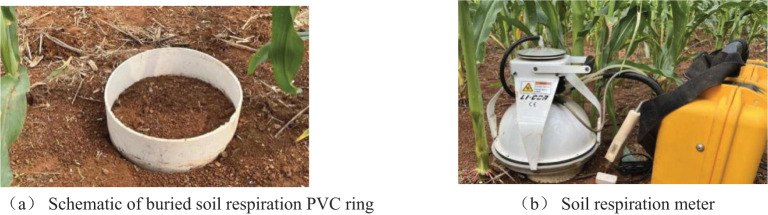
**(A, B)** Schematic diagram of soil respiration measurement.

Soil respiration was determined using a Li-6400 portable gas analysis system (Li-Cor Inc, NE, USA) connected to a Li-6400-09 soil respiration chamber (**Figure 3**). In the experiment, two PVC rings were installed in each replicated plot, and two plants with uniform growth near the center of each row were selected and inserted into the PVC ring (inner diameter of 10.2 cm, height of 5 cm) at 1/2 plant spacing or at the same time at a distance of 5 cm from one of the plants, with an insertion depth of 2 cm. All visible plants and animal life inside the PVC ring should be removed before measurement to ensure that the measurement results reflect the biological activities within the soil system and not the respiration of foreign organisms. The soil respiration rate of each plot was the average of two cycles of the instrument, and each cycle was approximately 4–5 min. The seasonal variation of soil respiration of all treatments was measured between 07:00 and 09:00, and related studies have shown that the soil respiration rate measured at this time can represent the average value of the day (Baldocchi et al., 2018). Measurements were taken every 10 days during the maize growth cycle and were delayed when heavy rainfall occurred.

##### 2.3.1.2 Soil oxygen content

Using an oxygen meter (MO-200 Oxygen Meter, USA), oxygen content was measured at 20 cm below the soil surface concurrently with soil respiration rate measurements. The measurements were conducted on the same dates and times as the soil respiration rate measurements. Oxygen sensors were placed inside porous plastic chambers and positioned in the soil.

##### 2.3.1.3 Aerated soil porosity

The soil air-filled porosity was calculated based on measured soil moisture content, as indicated in Equation 3. Soil moisture content was automatically measured by field moisture sensors (Melsted et al., 1949):


(3) $$P_{a}=\frac{\rho_{s}-\rho_{b}}{\rho_{s}}-\theta$$

In the equation, $P_{a}$ represents soil air-filled porosity, expressed as a percentage (%), $\rho_{s}$ denotes particle density, measured at 2.6 g·cm^−3^; $\rho_{b}$ is the soil density, 1.65 g/cm^3^; θ represents a soil water content of 0–30 cm, expressed as a percentage (%).

#### 2.3.2 Soil fertility

Almost all physiological metabolic processes in soil are related to soil microorganisms. Bacteria constitute approximately 94% of the soil microbial community, with actinomycetes and fungi comprising the remaining 4% to 5%. Changes in soil enzyme activity reflect variations in soil microbial quantity and diversity, soil organic matter status, soil aeration, temperature, moisture, pH, and other environmental factors (Jin et al., 2024). Therefore, this study selects soil enzyme activity and bacterial biomass as indicators to evaluate soil fertility.

##### 2.3.2.1 Bacterial biomass of soil, BAC

Soil samples were collected using an S-shaped multi-point sampling method from the top 15 cm of soil in the crop growth area. Samples were taken from the plow layer at depths of 0–10 cm, 10–20 cm, and 20–30 cm, respectively. Fresh soil samples were collected after removing impurities such as stones and plant residues and were thoroughly mixed by layers. Five sampling points were selected per experimental plot to collect soil samples. Soil bacterial biomass was quantified using the plate count method (Lian, 2023; Li et al., 2024). The sampling date was consistent with the date of measurement of indicators such as soil respiration.

##### 2.3.2.2 Soil enzyme activities

Soil enzyme activities were evaluated after collection of soil samples. Urease (URE) activity was determined using the phenol-hypochlorite colorimetric method. Catalase (CAT) activity was measured using the KMnO_4_ titration method. Soil phosphatase (PHO) activity was assessed using the p-nitrophenyl phosphate colorimetric method (Lei et al., 2023b; Zhuang et al., 2024).

#### 2.3.3 Root growth

**Figure 4:**
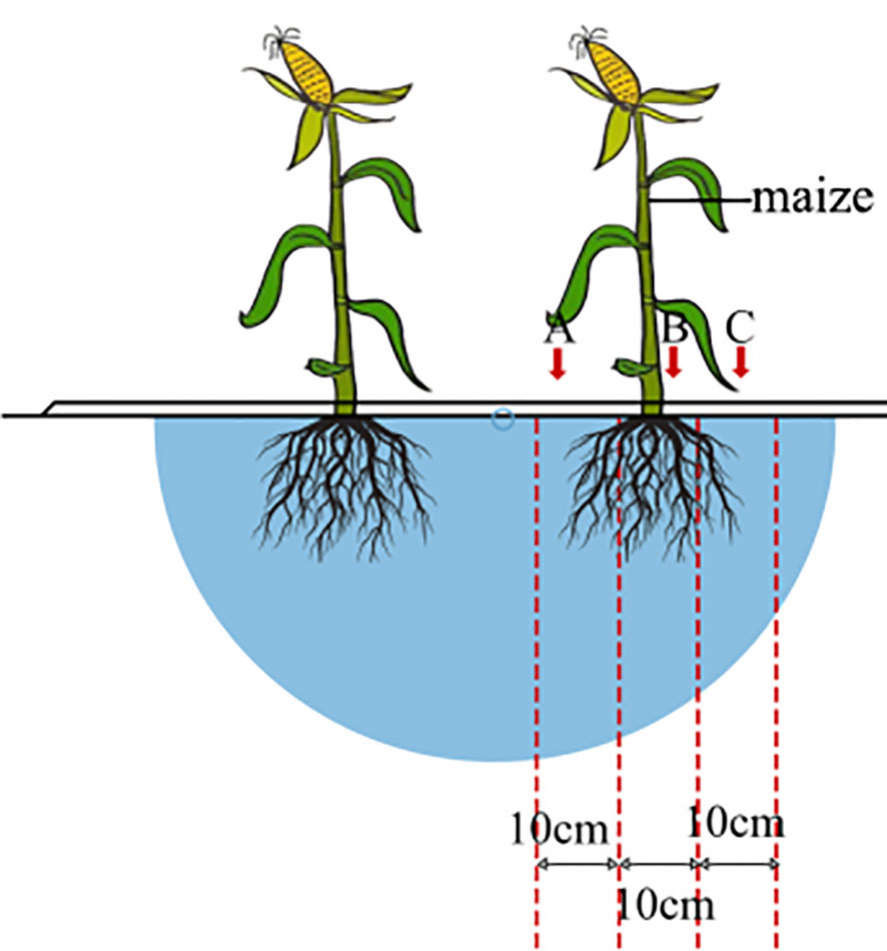
Schematic diagram of maize root collection structure under aerated percolation irrigation planting mode. Points A, B, and C indicate the location of the maize root sample collection site.

Root growth was evaluated by the root morphology and root dry weight in different treatments after maize harvest. Using the segmented soil auger method, three maize plants of uniform growth and in close proximity to each other were cut close to the ground, and root samples were collected from different lateral locations and layers of the maize at the base of the maize (point B in **Figure 4**), at a point 10 cm near the base of the maize on the side of the soakaway zone (point A in **Figure 4**), and at a point 10 cm far away from the base of the maize on the side of the soakaway zone (point C in **Figure 4**), and the roots were completely removed from the maize at a depth of 60 cm from the plant. The root system was completely dug out at a depth of 60 cm from the plant after removing stubs and grass roots. The roots were cleaned with a 400-μm sieve, scanned using a Perfection V700 scanner (Epson Inc., China) to obtain the root growth indexes, placed in an oven at 105°C for 15–30 min, and dried completely at 75°C.

#### 2.3.4 Growth physiology characteristics

##### 2.3.4.1 Plant height

The height of maize seedlings was measured using a ruler in millimeters. The stem height was measured from the bottom of the seedling to the highest growing node.

##### 2.3.4.2 Stem thickness

Stem diameter of maize plants was measured at 5 cm below the soil surface using a digital caliper (SATA Tools Co., Ltd, Shanghai, China).

##### 2.3.4.3 Photosynthetic properties

Photosynthetic parameters of maize were measured using a CIRAS-3 apparatus. Sampling was conducted at 10:00 a.m. Three to five matured and well-expanded leaves from the top of the maize plant were selected for measurements. In this process, we tried to avoid the damages for major veins, leaf edges, and diseased areas to minimize errors Measurements included chlorophyll content (Chl), net photosynthetic rate (Pn), and transpiration rate (Tr).

#### 2.3.5 Maize yield

Yield was measured using the “area method” during maize maturity time each year. A 60-m² area was randomly selected within each experimental plot. All maize ears within the selected area were harvested, and their total number and weight were collected. Subsequently, the total yield of the entire field was extrapolated based on these measurements (Yu, 2020).

### 2.4 Data analysis

All statistical analyses (including correlation analyses) in this paper were performed using SPSS statistical software 22.0, and statistical tests were performed using a significance level of 0.05 as a criterion for judgement, and structural equation modeling (SEM) was constructed using Smart PLS 3.0 software, by setting the independent variables (soil aeration and fertility), the mediating variable (root growth indicators), and the dependent variable (maize yield). The model was first validated by measuring the model (indicator loading, Cronbach’s alpha, combined reliability, and mean extracted variance) to ensure the reliability and validity of the data, and then tested the significance of the coefficients of each pathway through 500 samples using the Bootstrap technique to analyze how soil aeration and fertility indirectly affect maize yield through root growth indicators. Finally, in order to deal with possible Type I errors due to conducting multiple hypothesis tests, the Bonferroni correction method was used to adjust the significance level and thus the rigor of the results of the study.

## 3 Results

### 3.1 Soil environmental indicators

#### 3.1.1 Soil aeration

**Figure 5:**
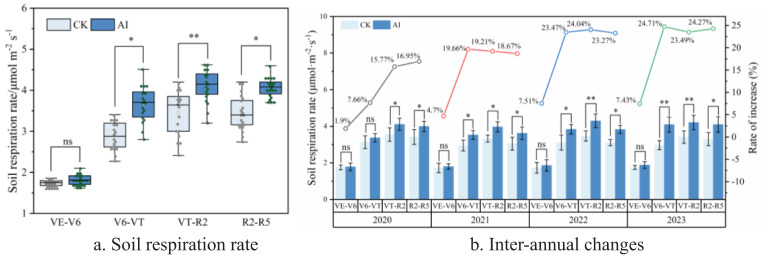
**(A, B)** Response of soil respiration rate to aerated irrigation technique.

**Figure 6:**
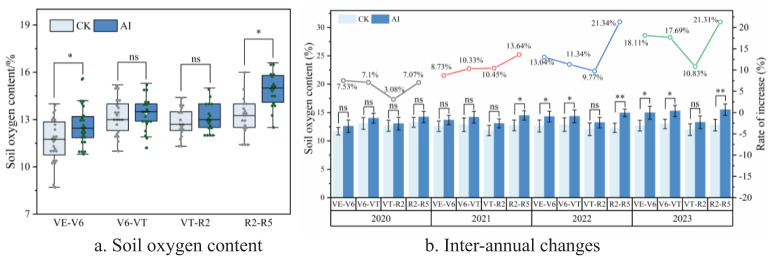
**(A, B)** Response of soil oxygen content to aerated irrigation technique.

**Figure 7:**
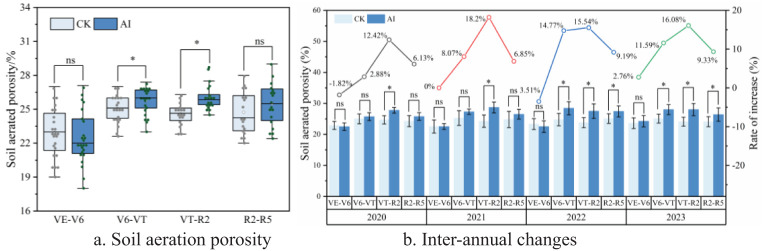
**(A, B)** Response of soil aerated porosity to aerated irrigation techniques.

Long-term AI technology had significant and highly significant effects on soil oxygen content and soil respiration rate, respectively (**Figures 5**, **6**). Compared with the CK treatment, the increases were 1.90% to 24.71% (*p* < 0.05) and 3.08% to 21.34% (*p* < 0.01), respectively. Although the AI technique can improve soil aerated porosity to a certain extent, only some of the measured sites showed significant differences (*p* < 0.05) (**Figure 7**).

Soil respiration rate showed a “single-peak” trend of “first increasing and then decreasing” throughout the maize reproductive period (**Figure 5A**). In the maize VE period, low soil temperature (**Figure 1**), high soil humidity, relative low microorganisms level in the soil, low soil respiration rate, and slow decomposition rate showed in the results. No significant difference was found between different treatments (*p* > 0.05). Soil oxygenation improved with aerated seepage irrigation technology (**Figure 6B**) and showed significant and highly significant differences compared to the CK treatment. Maize was in the period of tasseling to the filling at the VT–R2 period that the growth rate reached the peak and the oxygen demand and respiration rate of the root system were maximized. The mean value of soil respiration rate under aerated infiltration treatment reached 4.15 μmol m^−2^·s^−1^ (**Figure 5A**). The results of soil respiration rate changes under different treatments from 2020 to 2023 showed that the effect of AI increased year by year at all fertility periods as the year progressed (**Figure 5B**). In the VE–V6 period, the effect of AI on soil respiration rate was small, and although soil respiration rate increased under the AI treatment, none of the increases was significant. When maize entered the V6–VT and VT–R2 periods, the increases ranged from 7.66% to 24.71% and 15.77% to 24.04%, respectively, compared with the CK treatments (**Figure 5B**), and the effect was significant and enhanced year by year, and reached highly significant differences (*p* < 0.01) in 2022 and 2023.

Soil oxygen content under different treatments showed a trend of “gradual decline” throughout the reproductive period of maize (**Figure 6A**), and there was no significant difference in soil oxygen content between different treatments during the two periods of maize V6–VT and VT–R2 (*p >* 0.05), which is mainly due to the fast growth of maize and enhanced soil respiration rate. From 2020 to 2023, soil oxygen content under AI treatment was higher than that of CK treatment in all fertility periods (VE–V6, V6–VT, VT–R2, and R2–R5), and the increase year by year as the year progressed (**Figure 6B**), but in the VT–R2 stage, the enhancement effect of AI on soil oxygen content was enhanced year by year, and the increase was 7.10% to 17.69% compared with CK treatment (**Figure 6B**), but the overall increase did not reach a significant level (*p* > 0.05). In the R2–R5 stage, AI could significantly enhance soil oxygen content, and the increase reached highly significant levels (*p* < 0.01) in 2022 and 2023.

The effect of long-term AI technology on soil aerated porosity is shown in **Figure 7A**; in the VE–V6 period of maize, aerated infiltration irrigation technology instead decreased soil aerated porosity (**Figure 7A**); in the late maize growth period, aerated infiltration irrigation under the treatment compared to the CK group can improve soil aerated porosity to a certain extent, with an increase ranging from 2.76% to 18.20% (**Figure 7B**); and only some of the measurement points have significant (*p* < 0.05) and highly significant (*p* < 0.01) differences. From the data from 2020 to 2023, the technology can significantly increase soil aerated porosity during the V6–VT and VT–R2 periods (*p* < 0.05), and the improvement of soil aerated porosity by aerated infiltration technology is significant year by year over time, with gradual improvement of the soil structure and continuous improvement of soil aerated porosity (**Figure 7B**).

##### 3.1.2 Soil fertility

**Figure 8:**
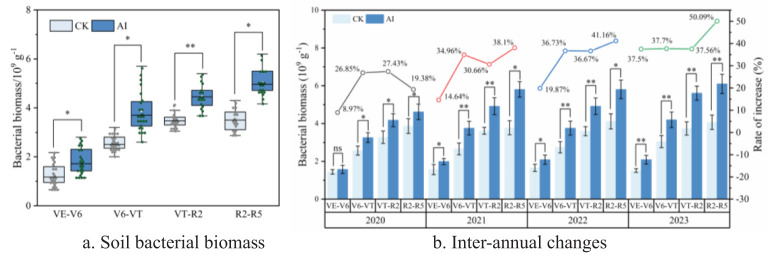
**(A, B)** Response of soil bacterial biomass to aerated irrigation techniques.

During the reproductive period of maize, soil bacterial biomass under different treatments showed a trend of “gradual increase” with the growth of maize (**Figure 8A**) ranging from 1.27 to 5.12×10^9^ g^−1^. The soil bacterial biomass under AI was significantly increased by 8.97%–50.09% compared with the CK group and had a significant effect (*p* < 0.05) on the growth of maize throughout the reproductive period (*p* < 0.05). In the later period (VT–R2 and R2–R5), the increase of soil bacterial biomass by AI treatment was more significant and increased year by year (**Figure 8B**). There were highly significant differences between different treatments (*p* < 0.01) when the atmospheric temperature and humidity were higher (**Figure 1**). The decomposition of soil organic matter was more active and the respiration rate of the crop root system reached the peak (**Figure 5A**). The warm and moist soil environment and the carbon source and nutrients released from the decomposition of organic matter provided rich nutrients for the growth of bacteria in the soil (Baldocchi et al., 2018), the bacterial biomass of maize R2–R5 reached the peak, and the increase of AI treatment compared with the CK group could reach 50.09% in 2023 (**Figure 8B**).

**Figure 9:**
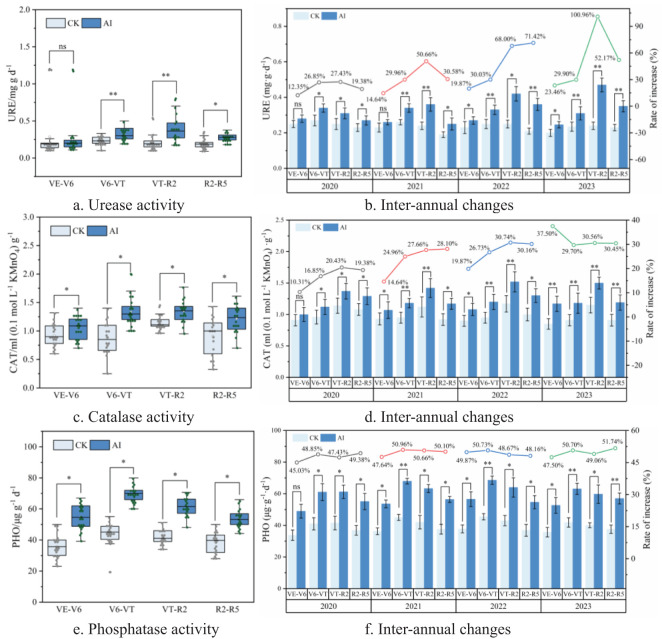
**(A–F)** Response of soil enzyme activities to aerated irrigation techniques.

Soil enzymes continuously provide nutrients and energy to the soil by promoting organic matter decomposition, mineralization, and recycling (Xu et al., 2024). The increases in URE, CAT, and PHO activities over the control were 12.35%–100.96%, 10.31%–30.74%, and 45.03%–51.71%, respectively. With the growth of maize, the more significant is the effect on soil enzyme activities, especially in the VT–R2 period of maize, the rate of nutrient cycling in the soil was accelerated, and the activities of these enzymes in the soil increased to reach the peak value. All of the above factors lead to the decline of soil enzyme activities (**Figures 5C, E**, **9A**) with the reduction of nutrient uptake by maize, the microbial activity is weakened, and the rate of decomposition and mineralization of organic matter is reduced when maize was in the R5 period. The activities of all three enzymes were significantly increased by AI treatments, and the long-term application of AI had a stabilizing and promoting effect. Among them, in the late maize growth period (VT–R2 and R2–R5), the increase of soil URE activity by AI treatment was significant; in particular, in 2023, the increase of URE activity in the VT–R2 period reached 100.96% (**Figure 9B**), and the increase of PHO activity reached 51.74% (**Figure 9D**); moreover, the peroxidase activity under AI conditions increased by 30.74% (**Figure 9D**) and 30.56% (**Figure 9D**) in 2022 and increased by 30.74% (**Figure 9D**) and 30.56% (**Figure 9F**) in 2023, respectively.

### 3.2 Root growth

**Figure 10:**
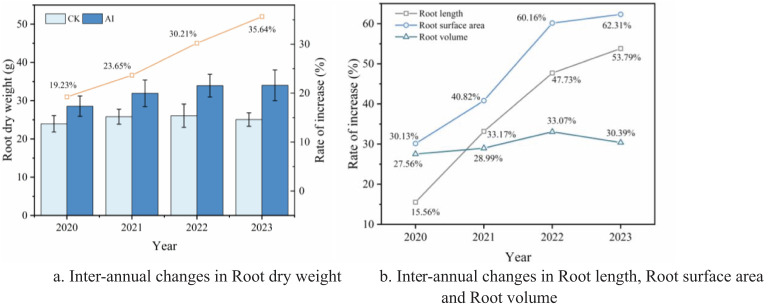
**(A, B)** Response of root indicators to aerated irrigation techniques.

Morphological plasticity of crops refers to the ability of crops to adapt to environmental conditions by changing the morphology and structure of the root system, stem, leaves, flowers, and fruits and other tissues under specific environmental conditions (Palada et al., 2010; Yu, 2023). As shown in **Table 1**, there were significant (*p* < 0.05) and highly significant (*p* < 0.01) promotion effects on root morphology indexes at different levels of soil after long-term aerated percolation irrigation. The effect on root length, root surface area, and root volume in the 15–30 cm soil layer was more significant under the AI technique (**Table 1**). Data from 2020 to 2023 showed that the AI technology showed consistency and stability across years. Soil depth root length, root surface area, and root volume increased by 15.56% to 53.79%, 30.13% to 62.31%, and 27.56% to 33.07%, respectively, using AI technology (**Figure 10B**), which indicated that aerated maize root maize under the percolation irrigation treatment could expand the root system deeper and more widely, improve the contact area between the root system and the soil, and provide a stable nutrient supply for maize during the critical period of maize growth. AI technology further increased root dry weight by improving root morphology indexes, with increases ranging from 19.23% to 35.64% compared with the CK treatment (**Figure 10A**).

**Table 1:** Effects of different treatments on morphological indicators of maize in different soil layers.

Year	Morphological characteristics	0–15 cm		15–30 cm		30–45 cm		45–60 cm	
		CK	AI	CK	AI	CK	AI	CK	AI
2020	Root length (cm)	708.40	833.76	233.71	272.71	212.52	251.81	141.68	165.86
	Root surface area (cm^2^)	225.02	294.13*	344.62	448.45*	87.16	114.08*	66.91	85.10*
	Root volume (cm^3^)	4.68	5.81	10.31	13.16*	1.38	1.63	0.65	1.11**
2021	Root length (cm)	694.50	966.45*	236.13	315.20*	216.31	277.41	143.80	194.96*
	Root surface area (cm^2^)	223.55	318.04*	344.49	490.52*	87.74	118.93*	63.22	85.11*
	Root volume (cm^3^)	5.18	7.53**	10.42	14.04*	1.30	1.52	0.42	0.67*
2022	Root length (cm)	778.52	1,050.31*	256.90	379.05*	233.75	320.34*	155.70	250.02**
	Root surface area (cm^2^)	253.65	406.47**	395.47	610.17**	100.73	160.28**	73.25	131.20**
	Root volume (cm^3^)	4.92	6.56	10.64	14.28*	1.31	1.82*	1.09	1.24
2023	Root length (cm)	806.85	1,239.31*	249.81	384.19**	231.09	320.91*	162.79	286.31*
	Root surface area (cm^2^)	272.87	454.66**	424.19	701.23**	108.04	164.99**	87.95	128.63*
	Root volume (cm^3^)	4.70	6.12*	9.46	12.50*	1.40	1.80*	1.51	1.85*

*Indicates a significant difference between group comparisons (p < 0.05), **indicates a highly significant difference between group comparisons (p < 0.01).

### 3.3 Growth physiology characteristics

#### 3.3.1 Agronomic indicators

**Figure 11:**
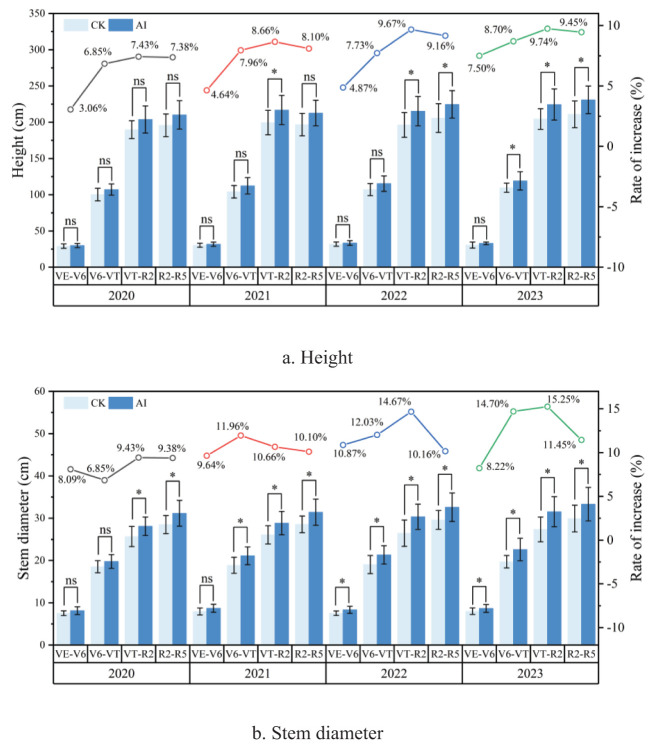
**(A, B)** Effect of aerated percolation technology on agronomic traits of maize.

The most intuitive effects of the different treatments were manifested in the morphological indicators of the crop, such as plant height, stem thickness, and leaf area. AI treatment could significantly increase maize plant height and stem thickness by 3.06% to 9.74% and 8.09% to 15.25%, respectively (**Figure 11**). It also indicated that aerated infiltration irrigation technology could increase the growth rate of maize plants, and that V6–VT was the critical period for maize growth. AI technology significantly increased maize plant height and stem thickness during 2020–2023 (**Figure 11**). With experimental years increased, the effect of AI treatments on maize plant height and stem thickness increased year by year with the most significant increase during the maize VT–R2 period in 2023. Plant height and stem thickness increased 9.74% and 15.25% compared with CK treatment (**Figure 11**).

#### 3.3.2 Photosynthetic properties

**Figure 12:**
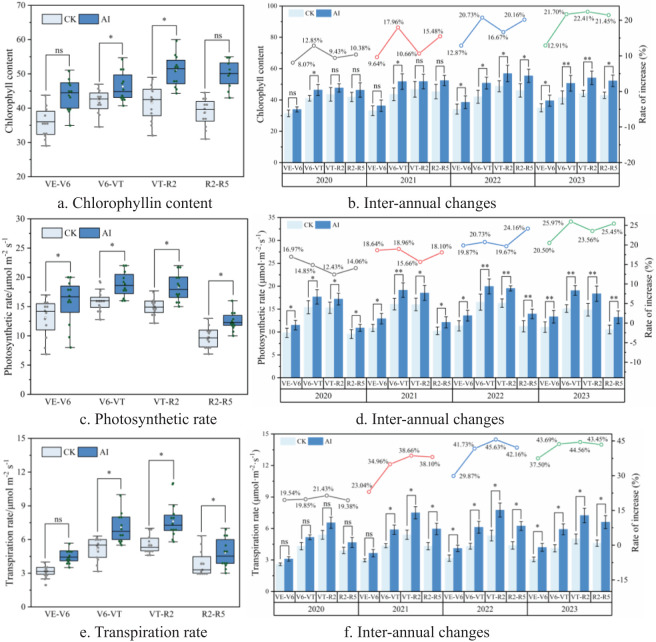
**(A–F)** Effect of aerated infiltration technology on the physiological characteristics of maize.

As the central pigment of photosynthetic reaction, chlorophyll plays the dual role of capturing light energy and separating charge, and at the same time, it can promote the opening of crop leaves to a certain extent, which is conducive to increasing the raw material (CO_2_) for photosynthetic reaction (Guo et al., 2023). The aerated treatments in this experiment all significantly increased maize chlorophyll content, photosynthetic rate, and transpiration rate by 8.07% to 22.41%, 14.06% to 25.97%, and 19.54% to 45.63%, respectively (**Figure 12**). The increases in chlorophyll content, photosynthetic rate, and transpiration rate were significant at several fertility stages in 2022 and 2023, and the boosting effect increased with each year. Among them, the increase in chlorophyll content reached 21.70% and 22.41% in 2023 at maize V6–VT and VT–R2 stages, respectively. At the maize V6–VT stage in 2023, AI treatment could significantly increase by 25.97% compared to CK treatment. The transpiration rate of maize under AI technology increased significantly in 2022 and 2023, especially at maize V6–VT and VT–R2 stages, which could increase up to 43.69% and 45.63% compared with CK treatment.

### 3.4 Maize yield

**Figure 13:**
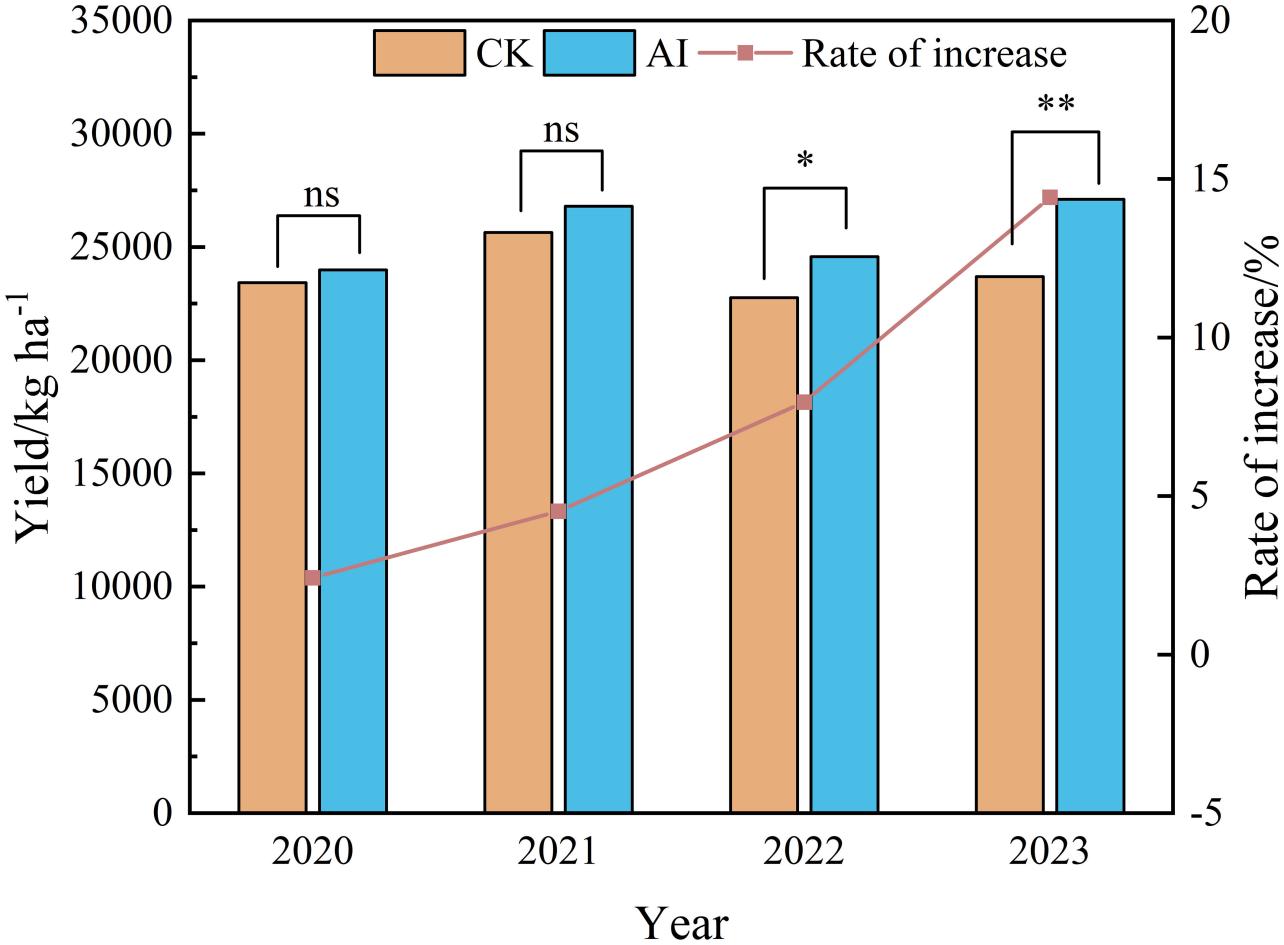
Effect of different treatments on maize yield.

The effect of different treatments on maize yield is shown in **Figure 13**. Under AI treatment, maize yield increased by 1.16% to 14.42% compared to CK treatment. It can also be observed that the yield-enhancing effect of AI treatment varied across different years. From 2020 to 2023, the enhancement in maize yield under AI treatment showed an overall increasing trend over time. This indicates that the promotion effect of AI treatment on maize yield gradually strengthened in the long-term experiment, reaching an increase of 14.42% in yield by 2023.

### 3.5 Analysis of yield increase mechanism of dry field maize under aerated irrigation mode

In this paper, field data from 2020 to 2023 were selected and analyzed by using Smart PLS 3.0 to analyze the structural equation model proposed in this study, and Bootstrap was carried out for the path coefficient test. The number of selected cycle test was 500 times. Before the analysis of the structural model, this paper launched the analysis of the measurement model, and the reliability test results of this study are good. All the indicators related to the latent variables can represent the concept itself better that the factor loadings are all greater than the threshold value of 0.7. On the other hand, the degree of consistency of the indicators (measurement items) is related to the content of the measurements. The higher the value of Cronbach’s alpha coefficient, the stronger the intrinsic consistency. Cronbach’s alpha values of the latent variables in this model were all greater than the threshold value of 0.7. The consistency between the items was good (**Table 2**). The construct validity of the latent variables is mainly divided into convergent validity and discriminant validity, which can be determined by composite reliability (CR) and average variance extracted (AVE), in which CR needs to be not less than 0.6, and AVE needs to be more than 0.5. The CR is greater than 0.7, and the AVE is greater than 0.7 in the model of this study. The Cronbach’s alpha of this model is greater than the threshold of 0.7. The results showed that the model has good reliability and validity and is suitable for subsequent SEM analysis.

**Table 2:** Measurement model test results.

	Index	Factor loadings	Cronbach’s alpha	Combinatorial reliability (CR)	Average extraction variance (AVE)
Soil aeration	a_1_ Soil respiration rate	0.801	0.499	0.703	0.732
	a_2_ Soil oxygen content	0.944			
	a_3_ Soil aerated porosity	0.693			
Soil fertility	b_1_ Bacterial biomass	0.950	0.783	0.897	0.768
	b_2_ URE	0.811			
	b_3_ CAT	0.791			
	b_4_ PHO	0.824			
Root growth	c_1_ Root length	0.702	0.783	0.878	0.713
	c_2_ Root surface area	0.729			
	c_3_ Root volume	0.913			
	c_4_ Root dry weight	0.856			
Growth physiology characteristics	d_1_ Height	0.803	0.748	0.771	0.746
	d_2_ Stem thickness	0.618			
	e_1_ Chlorophyllin content	0.913			
	e_2_ Photosynthetic rate	0.813			
	e_3_ Transpiration rate	0.885			
Yield	Yield	0.504	0.504	1	1

**Figure 14:**
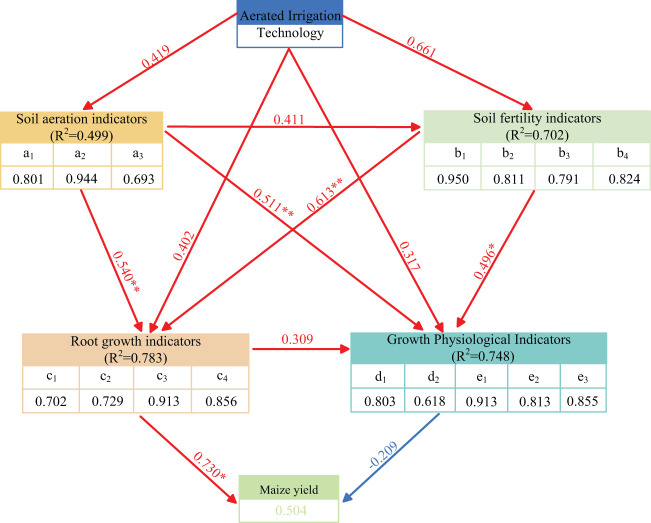
Structural model of maize yield increase by aerated irrigation technology.

The maize yield enhancement mechanism model under AI mode is illustrated in **Figure 14**. The results indicated that AI technology effectively explained the variance in soil aeration (*R* ^2^ = 0.499) and soil fertility (*R* ^2^ = 0.702). Specifically, the path coefficient from soil aeration to root metrics is 0.540 (*p* < 0.01), and soil fertility indirectly influences crop yield through root metrics with a path coefficient of 0.613 (*p* < 0.01). Causal relationship testing between soil aeration and soil fertility reveals a strong positive effect of soil aeration on soil fertility (path coefficient = 0.411, *p* < 0.05), which indicated that improved soil aeration enhances soil fertility, making nutrients more accessible and usable by plants. Enhanced soil fertility directly promotes maize yield increase and facilitates maize growth and development.

AI technology primarily improves soil aeration and soil fertility indicators, which provide a favorable growth environment for maize roots. Regarding indirect effects, soil aeration influences maize root metrics through its impact on soil fertility indicators. The effect of soil aeration on root metrics leading to yield is calculated as 0.540 × 0.730 = 0.394, while the effect of soil fertility on root metrics leading to yield is 0.613 × 0.730 = 0.447. This indicates that soil fertility has a direct and significant positive effect on maize growth and yield and strengthened through its indirect impact via root metrics.

The SEM structural equation model indicates that soil aeration and soil fertility have highly significant and significant effects on maize growth physiological indicators with path coefficients of 0.511 and 0.496, respectively. This suggests that soil aeration has a considerable impact on maize growth physiological indicators. Regarding indirect effects, soil aeration influences maize growth physiological indicators through its impact on soil fertility indicators. The effect of soil aeration on soil fertility leading to growth physiology is calculated as 0.411 × 0.496 = 0.204. AI technology not only promotes maize growth by providing oxygen directly and improving root environment, but also indirectly supports maize growth by enhancing soil fertility. Soil fertility acts as a mediator by influencing soil nutrient supply, further supporting maize growth physiological indicators. However, maize growth physiological characteristics have a path coefficient of −0.209 on maize yield, indicating a negative impact of maize growth physiological characteristics on yield.

## 4 Discussion

### 4.1 Long-term effects of aerated irrigation on soil environment

#### 4.1.1 Soil aeration

Good soil aeration ensures an adequate supply of oxygen around crop roots and facilitates the timely removal of CO_2_ produced by root respiration into the soil. It is a critical indicator for assessing soil health and the crop growth environment, which is crucial for enhancing soil fertility as well (Xu et al., 2024). The results of this study demonstrated that AI treatments significantly increased soil oxygen content (**Figure 5A**) and respiration rate (**Figure 5B**) compared to the control group CK, which is consistent with findings from previous research. This enhancement is primarily attributed to more efficient metabolic activities of soil bacteria and other microorganisms, as well as maize roots (Yu, 2020; Li R. et al., 2023; DeBoer et al., 2024). In this study, AI treatment maintained a favorable soil oxygen level (**Figure 6A**), although soil respiration increased during the V6–VT and VT–R2 stages under AI treatment (**Figure 5B**). This ensured normal metabolic activities of soil microorganisms. As maize entered the R2–R5 stage, most of its growth and nutrient accumulation were completed. Plants started focusing on transferring photosynthetic products (such as carbohydrates) to the grains. Root activity and root exudation also decreased, which resulted in reduced microbial activity and organic matter decomposition rates. Consequently, soil oxygen content and respiration rate gradually stabilized and showed a declining trend (**Figures 6A, B**). Studies by Li et al (DeBoer et al., 2024). similarly demonstrate that enhancing soil oxygen content effectively promotes soil oxidation–reduction reactions, improving soil water and gas environments to facilitate crop water and nutrient absorption. Research by Zhu et al. and Bhattarai et al. further supports that AI treatments increase soil oxygen content by 124% and 183% compared to non-aeration treatments (Bhattarai et al., 2005; Torabi et al., 2013; Bhattarai et al., 2015; Pendergast et al., 2019; Zhu et al., 2020). This increased oxygen supply promotes microbial reproduction and activity in the soil; enhances organic matter decomposition, nutrient release, soil fertility, and biological activity; and ultimately boosts crop growth rates and yields.

Soil aeration pore size and soil water levels exhibit a reciprocal relationship. The study indicates that AI treatments did not significantly affect soil aeration pore size. During the maize VE stage, AI treatments reduced soil aeration pore size (**Figure 7A**). This effect primarily stemmed from AI treatments introducing not only air but also a considerable amount of water during irrigation at the maize VE stage. Excessive water might fill the soil pores with decreased soil aeration pore size. In the later stages of maize growth, the soil’s self-recovery capacity gradually restored the pore size to its original state although AI treatments improved soil aeration pore size to some extent compared to the CK group. Therefore, this influence was not statistically significant enough to be reflected in the data (**Figure 7B**).

In addition, it is known in this study that the effects of AI on soil respiration, soil oxygen content, and other indicators increased gradually with time during the long-term experimental process, which was mainly attributed to the gradual improvement of the soil structure, the enhancement of the root system and microbial activity, and the cumulative effect. AI gradually improves soil aeration by introducing air during the irrigation process (Yu, 2020). Over time, the soil structure, which was originally compact, becomes looser and more porous (**Figure 6B**), which makes it easier for air to penetrate deeper into the soil and increases the oxygen content of the soil. Secondly, with the prolongation of AI, the higher oxygen content in the soil promotes the metabolic activities of the root system and soil microorganisms, and their metabolic products will further improve the soil environment (**Figure 8B**), forming a virtuous cycle that leads to a continuous increase in soil respiration and oxygen content. Thus, these factors work together to make AI show more significant benefits in long-term applications.

#### 4.1.2 Soil fertility

Soil oxygen content, respiration rate, and aeration pore size actively participate in soil microbial activities and root metabolism processes, facilitating the rapid release of nutrients and enhancing the content of available nutrients in the soil. Results from this study show that AI treatments had no significant impact on soil bacterial biomass during the maize VE stage (*p* > 0.05). However, they promoted an increase in soil bacterial biomass during the V6–VT and VT–R2 stages (**Figure 8A**). This finding is consistent with previous research indicating that AI techniques can increase the quantity and activity of bacteria and other microbes in the soil compared to conventional irrigation. It could also accelerate organic matter decomposition, enhance nutrient release, and improve soil fertility levels (Ben-Noah and Friedman, 2018). Furthermore, studies on acidic forest soils have shown that increasing soil oxygen concentration significantly increases the number of nitrifying bacteria and accelerate the conversion of ammonium nitrogen to nitrate nitrogen (Li et al., 2022).

Soil hypoxia stress inhibits soil enzyme activities and microbial populations, affecting nutrient cycling and organic matter degradation (Li Y. et al., 2023). AI treatment significantly increased the activities of three soil enzymes compared to CK treatment (**Figures 9A, C, E**). URE and PHO can accelerate the decomposition of urea and organophosphorus compounds in the soil and increase the effectiveness of nutrients (Yu et al., 2022b), which are mainly affected by soil nutrient content, organic matter content, microbial activity and soil aeration. The V6–VT period of maize is the nutrient growth period when the demand of nitrogen reaches the peak. It is necessary to carry out additional sufficient nitrogen and phosphorus fertilizers at this stage. Good soil aeration under AI treatment further promoted the secretion and action of URE and PHO. Then, URE could effectively convert urea into ammonium nitrogen for plant uptake while PHO accelerated the mineralization of organic phosphorus to produce inorganic phosphorus that could be absorbed by plants (Han et al., 2006; Li et al., 2020).

During the R2–R5 stage when maize grow into the reproductive stage and focus on grain filling and maturation, root uptake ability, enzyme secretion, and activity experienced a slowdown (**Figure 9A**) due to changes in physiological requirements although the soil aeration was improved due to AI treatment (Zhu et al., 2008; Zhang et al., 2012). CAT is used in plants and microorganisms to decompose hydrogen peroxide and protect cells from oxidative damage, and the activity of CAT is closely related to the activity level of microorganisms in soil. Relevant studies have shown that the higher the microbial activity, the more enzymes are secreted and the activity is enhanced, which is consistent with the experimental results in this paper (**Figure 8**). Hydrogen peroxide, one of the metabolic by-products, has to be decomposed by CAT to prevent the damage of oxidative stress on microorganisms and plant cells. Therefore, the higher the microbial activity, the stronger the secretion and activity of CAT, which creates a positive feedback mechanism and has significant effects in the rapid growth period (V6–VT) of maize (**Figures 9C, D**).

Overall, AI treatments have a positive impact on soil microbial activity, organic matter decomposition, and nutrient release, especially on increases in soil bacterial biomass during specific growth stages. This acceleration of organic matter breakdown and nutrient release contributes to enhancing soil fertility levels, thereby providing a favorable soil environment conducive to healthy crop growth.

### 4.2 Long-term promotion effects of aerated irrigation on maize growth

Roots are the primary organism to absorb water and nutrients for maize growth (Wei et al., 2021; Yu et al., 2022b). AI treatments, aimed at improving soil conditions, initially affect the growth of maize roots. This study demonstrates that under AI treatment, root length, surface area, volume, and dry weight increased significantly by 15.56% to 53.79%, 30.13% to 62.31%, 27.56% to 33.07%, and 19.23 to 35.64%, respectively (**Table 1**). Consistent with many studies, the reasons can be analyzed from two main aspects. Firstly, as indicated in 3.1 Soil environmental indicators of this study, AI treatment enhances soil oxygen availability, creating a favorable environment for aerobic microorganisms (**Figure 8A**). This increase in microbial populations and soil enzyme activities (**Figure 9**), facilitated by organic matter decomposition, releases abundant nutrient elements such as nitrogen, phosphorus, and potassium, thereby providing ample nutrition for maize root growth and supporting the development of deeper roots (Li Y. et al., 2023). Secondly, enhanced root extension and activity improve maize’s capacity to absorb water and nutrients. The increased root length and volume expand the root–soil contact surface area to reach for greater absorption of water and nutrients from a broader soil area. This not only supports healthy aboveground growth, but also promotes further root growth and expansion (Zhuang et al., 2024) as was shown by the significant increase in root dry weight (**Table 1**). In addition, this study showed in a long-term experiment that AI technology had more significant effects on root length, root surface area, and root volume in the 15- to 30-cm soil layer (**Table 1**), partly due to the fact that the physical structure of soil in the 15- to 30-cm layer is relatively loose (Zhao et al., 2017), which is more susceptible to the positive effects of AI, and oxygen is more likely to penetrate and diffuse into the root zone, while the soil physical structure of the 30- to 60-cm soil layer is usually denser (Silwal et al., 2020; Yu et al., 2022b), with a relatively low porosity, and the higher degree of compaction restricts the diffusion rate of oxygen, making it difficult for oxygen to penetrate deeply into deeper soil layers. Even if AI treatment passes air into this layer, the diffusion efficiency of oxygen in deep soil is not as good as that in shallow soil, and the absorption capacity of the root system is inhibited, leading to its relatively small effect in the 30- to 60-cm soil layer.

On the other hand, the 15- to 30-cm soil layer is usually the main area for nutrient and water absorption by the maize root system, which is relatively dense and active (Yu et al., 2022a), and can cover a large area of the soil, and can effectively absorb water and nutrients in the soil, and the AI treatment improves the soil aeration of this soil layer and promotes respiration and metabolic activities of the root system, thus improving the growth capacity of the root system and enhancing the efficiency of water and nutrient absorption. The 30- to 60-cm soil layer belongs to the deep layer, the power of root expansion to the deep layer mainly comes from water shortage and other stress conditions, while the root system prefers to develop in the shallow layer under normal conditions. Therefore, the number of maize roots in the 30- to 60-cm soil layer is relatively small and the expansion speed of the root system is relatively slow and root density is low. Although AI treatment can improve soil aeration in this layer, root absorption capacity is limited and the effect is not significant due to less roots in this layer.

Maize roots absorb water, which is crucial for photosynthesis and transpiration in leaves and serves as the foundation for chlorophyll synthesis (Melsted et al., 1949; Zhu, 2020). Compared to the CK group, AI treatment increases chlorophyll content in maize leaves (**Figure 12A**), raises stomatal conductance, elevates intercellular CO_2_ concentration, and enhances transpiration rate (**Figure 12E**). Concurrently, maize photosynthetic rate also increases (**Figure 12C**). Similarly, Li et al (Ben-Noah and Friedman, 2018). found that adequate oxygen supply in soil benefits root growth and promotes aboveground photosynthesis. Research by Lei et al. indicates that aerated treatments significantly improve maize photosynthetic efficiency that is mainly attributed to enhanced root respiration facilitated by sufficient oxygen supply. This improvement in soil and root environment indirectly enhances aboveground photosynthesis and transpiration, thereby increasing root absorption capacity for water and nutrients, which, in turn, promotes aboveground growth and development (Li Y. et al., 2023). It is known that sufficient oxygen under AI treatment enhances root efficiency in water and nutrient uptake. This is transported upwards through root cell interstices or the vascular bundles of the stem to reach the leaves, ensuring ample water and nutrient supply to maize plants. Regulation of stomatal conductance adjusts water and gas balance to maintain internal leaf humidity and gas concentration (Li et al., 2020), thereby promoting chlorophyll synthesis and accumulation to support photosynthesis and transpiration in crop leaves. Moreover, studies also show that AI treatment not only increases maize chlorophyll content and enhances net photosynthetic rate (Han et al., 2006) but also delays leaf senescence, resulting in increased production of photosynthetic products.

This study also confirms that maize stem diameter increases rapidly during early growth under different treatments, with AI treatment showing significantly higher growth rates compared to the CK treatment (**Figures 8A, B**). As the maize growing season progresses, the growth advantage of AI treatment becomes increasingly evident. During the maize V6–VT stages, AI treatment significantly influences maize stem diameter at a very significant level (*p* < 0.01). This research aligns with previous findings in the development of AI technologies across various plant species, primarily influenced by factors such as cell elongation and division, vascular bundle growth, cell wall synthesis, and photosynthetic products. Long-term field trials and previous discussions in this study indicate that under AI technology, maize roots exhibit efficient water and nutrient uptake capabilities, providing ample nutrients to the stem through upward transport. This supports the synthesis of robust and thick cell walls in the maize stem (Xing, 2015), while carbohydrates produced through photosynthesis provide energy and material foundations for stem growth, thereby promoting maize growth and stem robustness (Xiao Z. et al., 2023).

### 4.3 Long-term maize yield mechanism under aerated irrigation technology

The soil environment improvement aims to improve crop yield. This study showed that aerated infiltration technology can alleviate the low oxygen stress of soil, which could promote soil respiration (Li et al., 2024), and positively affect the root growth and physiological characteristics of maize (Ouyang, 2018; Yu, 2020; Pang et al., 2023; DeBoer et al., 2024). The continuous 4a aerated infiltration technique significantly increased maize yield by 1.16% to 14.42% compared to the traditional infiltration technique (**Figure 13**). Because the planting situation needs to adapt to the new technology and conditions, it takes time to take full advantage of the technology. The initial yield increase effect of aerated infiltration is weak with the passage of time and adaptation. The advantages of the aerated infiltration technology include improved soil aeration and soil fertility, and the accumulation of the synergistic effect on maize yields. However, with the yield increase reaching the peak in 2021, there were unstable fluctuations in the late yield increase effect. On the one hand, it was due to the fact that climatic conditions in different years would have fluctuating effects on plant growth and yield. The maize jointing stage (V6)–tasseling stage (VT) (35–40 days after sowing) is the period of rapid growth of maize plants when stalks begin to elongate rapidly and most leaves unfold with a significant increase in water demand. Many studies have shown that the water demand in this stage accounts for 30%–35% of the water demand in the whole reproductive period. However, the low rainfall during this period in 2022 and 2023 (**Figure 1**) and the water deficit environment will lead to soil drought. Although AI treatment can help to improve the aeration of the root system, the water shortage still limits the growth of the crop resulting in a less than expected increase in yields. On the other hand, the high temperature of the weather in 2022 and 2023 (35°C) aggravated transpiration, and maize needed more water to maintain physiological activities. However, variations in precipitation and instability in soil moisture supply may cause plants to enter a state of water stress under high-temperature conditions, affecting flowering and kernel formation in the later stages of maize. In addition, the improvement of soil fertility under soil aeration conditions did not grow linearly (Jiang et al., 2024), such as fertilizer application rate, timing of fertilizer application, and soil type, which could lead to the fluctuation of soil fertility. The nutrient content of the soil may increase in a certain period of time but may fluctuate or decrease in another period. A combination of factors will directly affect crop growth and yield.

SEM was used to further evaluate the interactions and intensity of influence among soil aeration, soil fertility, and root indicators under AI treatment. The path coefficients were used to reflect the degree of influence of each variable on the other variables, which showed that AI technology directly affected soil aeration and soil fertility indicators with path coefficients of 0.419 and 0.661, respectively. The data in **Table 2** showed that soil respiration rate (0.801), soil oxygen content (0.944), and soil aeration porosity (0.693), with factor loading values higher than 0.7, indicate that the above measurements can well characterize soil aeration and that the AI treatment significantly enhances the supply of oxygen to the soil, especially by increasing the oxygen content of the soil, which strengthens the respiration rate of the soil (Yu et al., 2022a). It further promoted microbial decomposition activities, increased the rate of organic matter decomposition (Zhu, 2020; Yu et al., 2022a), and accelerated the ecosystem activity throughout the root zone. Bacterial biomass (0.950), URE (0.811), CAT (0.791), and PHO (0.824) in the soil fertility index system had high factor loadings, indicating that the AI technology significantly increased microbial activity in the soil, especially aerobic microbes. A large number of scholars’ studies have also shown that this technology accelerates the decomposition of organic matter to accelerate the mineralization process of key nutrients in the soil, such as nitrogen, phosphorus, and potassium, which enhances soil fertility and directly provides the basis for nutrient supply for root growth (Ouyang and Tian, 2023 2023; Shahzad et al., 2019; Li et al., 2024). Soil aeration index also had a significant positive effect on soil fertility index (path coefficient of 0.411), and this result also further proved that higher soil respiration rate and soil oxygen content under AI technology made microbial metabolism more active, which improved soil fertility (**Figure 14**).

This study examined that soil aeration and soil fertility had a significant positive effect on root indexes with path coefficients of 0.540** and 0.613**, respectively. In the process of root growth, soil fertility and aeration worked together to generate a positive feedback mechanism to enhance soil fertility by increasing root respiration and soil microbial activity. Soil fertility promotes root expansion by providing sufficient nutrients. The root system can carry out respiration efficiently and generate more energy for root cell growth and expansion. Root length, root surface area, and root volume were increased consequently. Although soil aeration has a direct effect on root growth, it is more important to promote root growth and expansion by improving soil microbial activity and promoting organic matter decomposition and nutrient release. Moreover, soil aeration could improve root growth and root biomass accumulation by increased soil composites.

A strong root system is the basis for high maize yield by acquiring more water and nutrients (Yu et al., 2022b), especially under drought or soil fertility deficit conditions. It can promote water and nutrient redistribution in the plant and maintain leaf photosynthesis and metabolic activities by enhancing synergistic interactions with the aboveground parts, thus mitigating the negative effects of drought and nutrient deprivation on the crop yield, and preventing the maize from yield reduction due to water deficit. The results of this study showed that root growth had a large effect on maize yield with a path coefficient of 0.730** (**Figure 14**). AI treatment can improve maize root growth (**Table 1**), which provides sufficient nutrients and water for the growth of the aboveground part. The nutrients can support maize reproductive growth by participating in cell division, photosynthesis, and metabolism processes of the maize growth process. In the meantime, it provides essential energy to support the healthy growth and high yield of maize.

However, this study found that physiological growth indicators of crops did not significantly affect crop yield (*p* > 0.05). The reason behind this could be attributed to excessive physiological activities, such as high photosynthetic efficiency (Jiang et al., 2024), which consume a substantial amount of energy and resources for the development of physiological characteristics (e.g., expansion of stems and leaves). Factors like leaf area, stem thickness, and plant height compete for limited water and nutrient resources, thereby affecting the formation, development, and final yield of maize grains (Palada et al., 2010; Baldocchi et al., 2018; Guo et al., 2023; Jin et al., 2024; Xu et al., 2024). As noted by Gliński in “Soil Aeration and Its Role for Plants”, plants possess a set of growth regulatory mechanisms that allow them to adjust growth and development processes based on external environmental conditions and internal needs. In situations of limited resources, the expansion of plant stems and leaves competes for limited water and nutrient resources (Waadt et al., 2022). When these resources cannot meet all growth demands, plants may prioritize the most critical aspects of growth, potentially at the expense of grain formation and development (Meena et al., 2019; Johnson et al., 2022).

## 5 Conclusion

In the dryland of Zhanjiang City, Guangdong Province, AI technology can improve soil aeration, provide a more suitable root growth environment for maize growth, and significantly improve maize growth characteristics and yield. We demonstrated that AI technology significantly improved soil aeration (path coefficient of 0.419) and soil fertility (path coefficient of 0.661), which promoted maize root growth (path coefficients of 0.540 and 0.613, respectively), and ultimately, the optimized root system directly contributed to the increase in maize yield (path coefficient of 0.730). Thus, the improvement of soil fertility in the soil root zone and the resulting enhancement of maize root growth by AI technology are the key to increased maize yield. Although growth physiological traits play an important role in the healthy growth of maize, their contribution to final maize yield is less direct and significant than that of root health due to their possible negative effects and higher resource consumption.

Overall, we will continue to conduct long-term experiments and introduce bio-analytical techniques to further assess the effects of AI on maize quality and nutrient composition. In addition, we will adjust the technical parameters by simulating different climatic conditions (e.g., prolonged droughts and continuous rains). The relationship among investment cost, operation cost, and fertilizer cost will be evaluated to achieve the optimal and sustainable AI technology that will be beneficial for agricultural productivity and environment protection.

## Data Availability

The original contributions presented in the study are included in the article/supplementary material. Further inquiries can be directed to the corresponding author.
